# Giant Extracranial Meningioma Associated With Hormonal Imbalances Due to Thyroidectomy: Case Report and Literature Review

**DOI:** 10.7759/cureus.51376

**Published:** 2023-12-31

**Authors:** Corneliu Toader, Bogdan-Gabriel Bratu, Razvan-Adrian Covache-Busuioc, David-Ioan Dumitrascu, Luca-Andrei Glavan, Alexandru Vladimir Ciurea

**Affiliations:** 1 Department of Neurosurgery, “Carol Davila” University of Medicine and Pharmacy, Bucharest, ROU; 2 Department of Vascular Neurosurgery, National Institute of Neurology and Neurovascular Diseases, Bucharest, ROU; 3 Department of Neurosurgery, Sanador Clinical Hospital, Bucharest, ROU

**Keywords:** gender prevalence, postoperative complications, radiotherapy, endocrine dysfunction, surgical resection, hypothyroidism, neuro-oncology, giant tumor, who grade iii, meningioma

## Abstract

Meningiomas represent a prevalent class of primary brain tumors, with malignancies such as World Health Organization grade III meningiomas posing significant clinical challenges due to their aggressive nature and potential for recurrence. This case report showcases the clinical journey of a 67-year-old female patient presenting with a giant malignant meningioma post-thyroidectomy, who unfortunately succumbed to postoperative complications. The report offers a comprehensive analysis of the tumor's clinical presentation, including its substantial size, which qualifies it as a 'giant' meningioma, and explores the patient's endocrine dysfunction as a possible contributing factor to her neurological pathology. In the broader context of meningioma management, the report synthesizes data from multiple studies, underscoring the higher incidence of such malignancies in post-pubertal women and the complexity of treatment modalities. Surgical resection remains the cornerstone of treatment, especially when combined with adjuvant therapies. The report concludes with a discussion on the persistent gaps in knowledge regarding the pathogenesis of giant malignant meningiomas and the need for further research, particularly concerning the role of endocrine dysregulation in their development. This case underscores the imperative for multidisciplinary approaches and individualized treatment strategies in the management of malignant meningiomas, with an emphasis on the intricate interplay between endocrine factors and tumor progression.

## Introduction

Meningiomas are among the most common types of benign brain tumors, originating from the meninges and spreading within the entire central nervous system (CNS) [1]. However, if left unidentified, their enlargement may lead to serious life-threatening complications. They are grouped into three grades, based on the World Health Organization (WHO) Meningioma Classification, as follows: grade I (Benign), grade II (Atypical), and grade III (Malignant) [2]. The latter is characterized by abnormal and increased cellular growth, resulting in a higher possibility of invasion and recurrence within the CNS than the other types. Additionally, WHO grade III meningiomas are classified as papillary, rhabdoid, and anaplastic subtypes, each presenting cell variations and thus histological modifications.

Meningioma’s prevalence is estimated at 97.5 cases per 100,000 people in the United States [3]. The risk of malignant meningiomas increases with age, sharply escalating after 65 years [4]. Studies have confirmed that exposure to high doses of ionizing radiation has also been correlated with an augmented prevalence of acquiring this disorder, even though radiotherapy is believed to slow tumor progression, this being possible only in the case of small-sized tumors [5]. Furthermore, a connection between meningiomas and abnormal hormone levels in patients’ bodies was confirmed [6]. Thus, post-pubertal women are thought to have a higher incidence of WHO grade III meningiomas compared to men, with a total patient survival of 2-3 years [7]. The Central Brain Tumor Registry of the United States (CBTRUS) shows more than twice the prevalence rate in females compared to males, with an age-adjusted rate of 8.36 per 100,000 person-years for females, while only 3.61 for males [8,9]. 

## Case presentation

A 67-year-old patient with a known endocrinological deficiency, a hypothyroidism following subtotal thyroidectomy surgery performed in 2019, which was neglected therapeutically, and with known neurological pathology, previously operated for malignant meningioma in 2021 due to diffuse headache associated with right hemiparesis, is brought to the emergency room in 2023 for diffuse headache of moderate intensity. On objective examination, the patient showed clinically normal lungs and cardiovascular assessment showed rhythmic heart sounds, blood pressure of 125/70 mmHg, and ventricular rate of 117 beats per minute. The patient presents orbital distress characterized by exophthalmos in the right eye. At the epicranial level of the parietal segment on the right side there was a hard, immobile tumour formation. The patient was conscious but hardly cooperative.

At the neurological examination, the patient was conscious, uncooperative, with a right hemiparetic posture, without a head roll, preserved oculomotricity and no nystagmus. Motor deficit was objectified by determining decreased muscle strength (according to the graded segmental muscle strength scale) on the right limbs - 1/5 MRC (Medical Research Council) upper limb and 3/5 MRC lower limb - with hypertonia in both of these limbs, walking was possible with bilateral support for very short distances. There was also a speech impairment of mixed transcortical aphasia (sequelae). Romberg examination was impossible.

To analyze the degree of hypothyroidism, a complete panel of thyroid hormone levels was performed which revealed elevated value of thyroid-stimulating hormone (TSH)=7.2 mU/L, associated with decreased thyroid hormones, total triiodothyronine (T3)=30 ng/dL and total thyroxine (T4)=3.2 mcg/dL at admission, highly indicative of the postprocedural thyroidectomy hypothyroidism.

**Figure 1:**
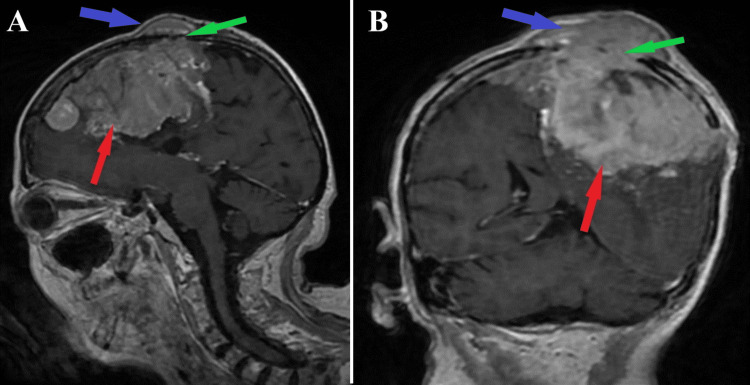
Preoperative MRI T1-sequence MRI T1-sequence sagittal section (A) and coronal section (B), both depict a giant (35x55 mm) malignant intracranial meningioma (red arrows) with important osteolytic skull lesions (green arrows) which determines extracranial invasion (blue arrows)

**Figure 2:**
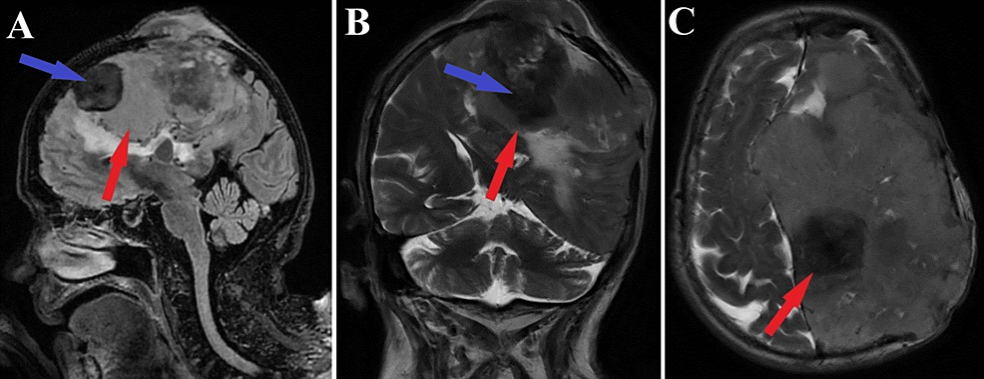
Preoperative MRI T2 and T2 FLAIR sequences Sagittal section of T2 sequence (A), coronal section of T2 FLAIR sequence (B), axial section of T2 FLAIR sequence (C). Red arrows indicate the giant malignant meningioma with various morphological aspects, while blue arrows highlight a calcification lesion. FLAIR: fluid-attenuated inversion recovery

**Figure 3:**
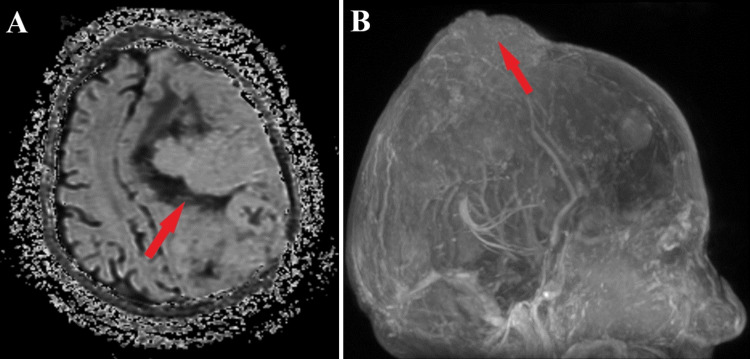
Preoperative ADC imaging aspect and 3D reconstruction Apparent diffusion coefficient (ADC) imaging scan (A) reveals the characteristic aspect of a malignant meningioma (red arrow), and a further 3D reconstruction (B) of MRI findings shows the extracranial morphological topography (red arrow).

An MRI showed the presence of parasagittal meningiomas on the left side, with a maximum diameter of 35 mm anterior-posterior and 55 mm cranial-caudal, numerous calcifications on the left frontal level with bone erosions showing mass effect and perilesional cerebral edema, which frames this malignant meningioma as a giant. Also, the ventricular system was displaced to the right of the midline by 10 mm, with sickle-shaped herniation, but without recent vascular lesions (Figures 1-3).

**Figure 4:**
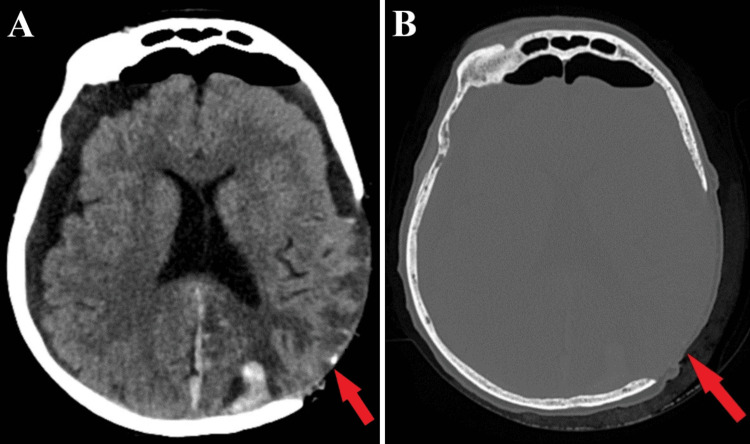
Postoperative CT scan aspect CT scan of tissue window (A) and bone window (B). In both images, the skull segment was not placed postoperatively (red arrows) due to significant compressive effects.

Preoperatively, the patient received hormone replacement therapy for hypothyroidism until normal range values were obtained. Regarding giant extracranial malignant meningioma, the treatment of choice was surgical resection, a gross total tumor resection being achieved. Due to the high risk of postoperative cerebral edema and multiple associated complications arising from the significant intracranial pressure effects, postoperatively, the craniotomy skull segment was not fixed (Figure 4).

**Figure 5:**
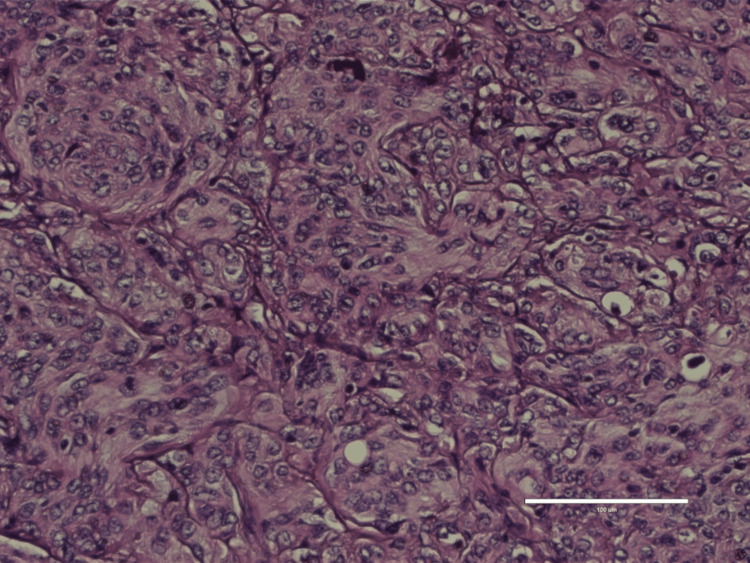
Histopathology results of meningioma Figure depicts an anaplastic meningioma with characteristic sarcoma-like changes and significant mitotic activity higher than 25 mitosis/10 high-power fields, 100X

**Figure 6:**
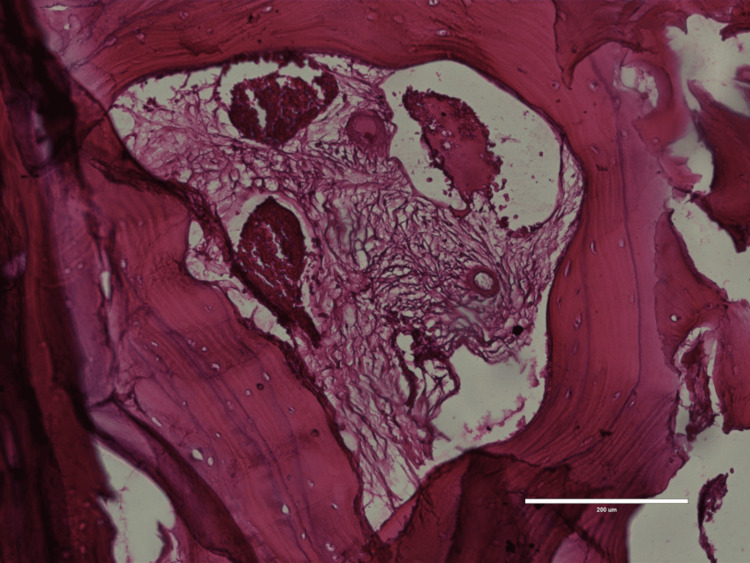
Histopathology examination of skull fragment Image shows skull invasion of the meningioma at the level of cortical bone, 200X

Histopathology examination of the tumor process (Figure 5) and skull fragment (Figure 6), revealed sarcoma-like anaplastic changes and important mitotic activity higher than 25 mitosis/10 HPF (high power fields), suggestive of an anaplastic meningioma, with associated xanthomatous morphology and multiple calcification lesions.

**Figure 7:**
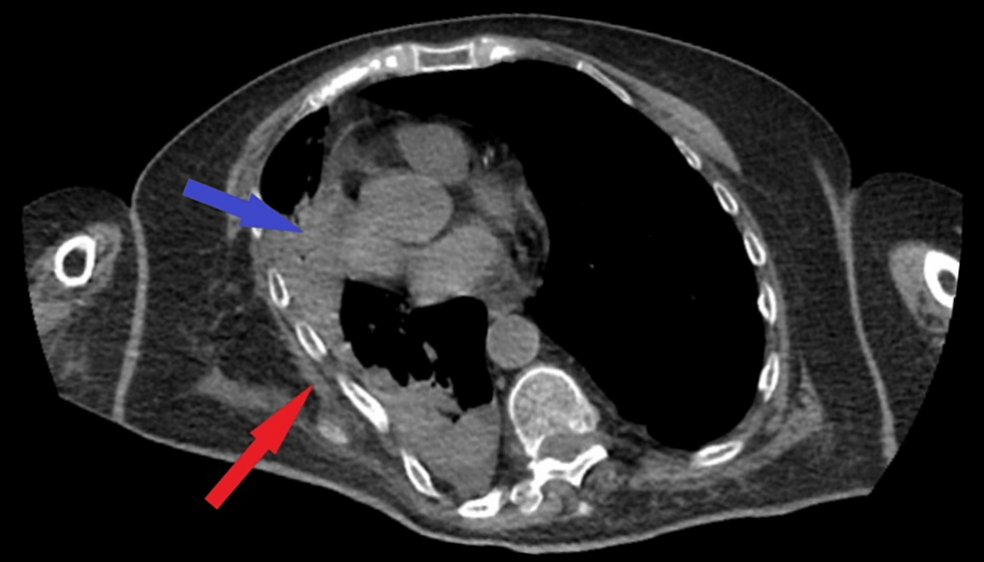
Postoperative pulmonary CT scan After 2 days postoperatively, collapse of the lungs was observed on CT scan consisting of anteriorization of rib arcs (red arrow) and atelectasis lesions (blue arrow)

Two days post-surgery, the patient exhibited respiratory distress. A pulmonary CT scan was performed, revealing pulmonary collapse with pleural involvement on the right side, anterior displacement of the rib arcs, and areas of atelectasis (Figure 7). Unfortunately, on the fifth day post-surgery the patient had a cardiac arrest due to pulmonary complications.

## Discussion

Malignant meningiomas can also be classified as per the size of the tumor. Thus, a WHO grade III meningioma could be classified as giant if it exceeds 5 cm in diameter [10]. In our case, the patient’s tumor had a considerably larger diameter, thus the condition is fulfilled. However, the specific mechanisms by which meningiomas are able to grow until they are considered ‘giant’ are still unknown.

Regarding the prevalence of this condition, several studies have highlighted the possibility of endocrine dysfunctions and hormone-based therapies to play an insightful role as risk factors for meningiomas [11,12]. Our patient underwent subtotal thyroidectomy surgery that led to hypothyroidism, a common endocrine condition in its field. Therefore, since giant malignant meningiomas were proven to be correlated with hormonal imbalances, our study suggests that the low levels of T3 and T4 may have led to our patient's neurological condition [13].

Furthermore, various cases reported a higher prevalence among female patients in acquiring this ailment; some of these studies thus emphasized the most common risk factors mentioned earlier (Table 1). As for the treatment of giant intracranial meningiomas, the gold standard is total resection of the tumor, which depends on the tumor’s size and location [14,15].

**Table 1:** Main surgical approaches in the case of gigantic malignant meningioma N = number of patients; F/M = female/male patients; GTR = total growth resection; STR = subtotal growth resection; PR = partial resection;

First autor & year	N	Sex		Extent of removal N (%)			Surgical approach	Postoperative complications N of pacients (%)			
		F	M	GTR	STR	PR		Headaches	Hemiparesis	Visual symptoms	Cranial nerve palsy
Sughrue et al 2009 [16]	1	1(100%)	-	1 (100%)	0 (0%)	0 (0%)	Decompressive surgery	-	-	1 (100%)	-
Sanai et al 2010 [17]	12	6 (50%)	6 (50%)	1 (100%)	0 (0%)	0 (0%)	Modified far-lateral approach	8 (67%)	4 (33.33%)	0 (0%)	0 (0%)
Xiao et al 2013 [18]	21	5 (23.8%)	16 (76.19%)	12 (57.1%)	8 (38,1%)	1 (4,8%)	Anterior transpetrous approach	18 (85.7 %)	0 (0%)	0 (0%)	1 (4.8 %)
Champagne et al 2018 [19]	12	10(83.33%)	2 (16.66%)	2 (16,66%)	3 (25%)	7 (58,33%)	Pterional craniectomy	6 (50%)	1 (8%)	4 (33%)	0 (0%)
Li et al 2020 [20]	150	38 (54.3%)	32 (45.7%)	75 (50%)	44 (29,33%)	31 (20,66%)	Fronto-orbital	0 (0%)	10 (6.66%)	5 (7.5%)	40 (28.6%)

Table 1 highlights that the predominance of male patients diagnosed with giant malignant meningioma is extremely rare, but it predominantly affects women. The five studies outlined in Table 1 show that the average ratio between the number of female and male patients is 2:1, in favour of women, who are more prone to this condition, as demonstrated in our study [16-20].

Furthermore, the GTR prognosis holds a higher value, favoring complete tumor removal [20]. The surgical approach differs from one study to another, but the postoperative complications are predominantly similar, the most common ones being headaches and visual symptoms.

Radiotherapy treatment shows intricate results regarding its safety and efficacy against meningiomas. The proportion of tumors reducing after radiotherapy ranges from 1% to 46%, with the majority of studies recording rates between 20% and 30%. [21,22]. However, increasing the ionizing radiation does show promising results against small-sized malignant meningiomas. Beyond 60 Gy (Gray), studies highlighted various approaches that reported successful local control, without significant adverse effects [23,24].

After all, available guidelines mention combined therapy, consisting of maximal safe resection and adjuvant radiotherapy, to be strongly recommended as a treatment strategy for malignant meningiomas [25,26]. Atypical meningiomas treated exclusively with resection, especially in cases with subtotal resection, revealed a significant tendency for recurrence. While some studies hinted at the promising advantages of adjuvant radiotherapy, they might not have had statistical significance because of the small sample sizes [27-29].

Moreover, a one-time Gamma Knife treatment with a peripheral dose of 12-14 Gy in one session has shown promising results for patients who can’t undergo craniotomy. Yet, this approach only works for tumors with an average diameter of less than 3-3.5 cm, thus preventing severe radiation-induced toxicity [30,31]. Since WHO grade III meningiomas have recurrent growth, adopting a therapeutic strategy that consists of repeated Gama Knife Surgery may lead to prolonged survival of patients [32].

Regarding the correlation between meningioma development and endocrinological disturbances, previous studies determined specific hormonal progression factors to interplay with limiting the meningioma development in women in the premenopausal phase [31]. When initially comparing thyroid disorders to meningioma, no statistically significant results were obtained, but an excess of pituitary function, being highly related to thyroid disturbances, might determine meningioma progression [33]. 

In a comprehensive analysis of differentiated thyroid cancer cases treated with radioiodine, Damle et al. identified five instances of symptomatic meningiomas necessitating surgical intervention among a cohort of 4,692 patients (comprising 1,524 males and 3,168 females). All five patients were females, with surgical histories including three total thyroidectomies, one hemithyroidectomy, and one subtotal thyroidectomy. Regarding postoperative conditions, one patient exhibited nodal disease in a whole-body diagnostic scan, whereas the remaining four presented only with remnant disease. Subsequent scans post-therapy revealed no additional anomalies. The average radioiodine dosage administered to these patients was 91.3 mCi, with a range of 40 to 208.8 mCi and a median of 55 mCi, as part of their routine ablation therapy. The temporal span between the onset of differentiated thyroid cancer (DTC) and the development of meningioma varied from 11 months to 7 years, averaging 3.5 years (median 4 years). Notably, all but one patient exhibited solitary meningiomas [34]. The prevalence of symptomatic meningioma requiring surgical intervention was found to be 106 cases per 100,000 patients with differentiated thyroid cancer [34].

In an intriguing case, meningioma was incidentally diagnosed during the radiotherapy workup for thyroid carcinoma. A 43-year-old male, post-total thyroidectomy for a 2.8-cm papillary thyroid carcinoma with local lymph node involvement, initially received 216 mCi I-131 as ablative therapy. Six months later, due to persistent iodide-avid disease in the neck, he underwent a second treatment with 262 mCi I-131. Post-therapy whole-body imaging revealed an angiomatous meningioma following I-131 administration for papillary carcinoma of the thyroid. The patient exhibited no neurological signs or symptoms. Interestingly, factors other than edema were suggested as significant for I-131 uptake in meningiomas, as magnetic resonance imaging revealed no substantial edema [35].

These findings align with broader literature, which has documented numerous instances of meningiomas following external radiation exposure [36,37].

Therefore, from the data presented above, we can underline a slight correlation and predisposition of this disease in female patients over 65 years old who have one or more dysfunctions of endocrinological origin [38]. Regarding a direct correlation between thyroidectomy-thyroid carcinoma radiotherapy and meningioma progression, it is still a well-debated subject in the current literature, and wider populations have to be taken into consideration to draw a clearer conclusion in this matter.

## Conclusions

In wrapping up our debate about this patient’s case, we have unearthed pivotal aspects regarding the prevalence of meningiomas. Our female patient has been diagnosed with a malignant meningioma, categorized among the giant tumors that are able to invade the CNS. We have delved into the multifaceted realm of neuro-oncology, revealing insights from other studies about the debatable prevalence and the intricated surgical approaches used to treat this condition. Thus, we have emphasized the high incidence of this type of tumor in women, especially the elder ones, and in patients with hormonal dysregulations.

Regarding the treatment for giant malignant meningiomas, combined therapy continues to be the gold standard of this approach. It’s essential to emphasize how, in the field of treatment approaches for these particular cases, surgery stands above radiotherapy. Thus, surgical interventions, particularly in combination with adjuvant treatments, hold a critical role in managing meningiomas and their impact on the CNS.

Our patient’s case highlighted for us a path to new ideas for further research: since she went through surgical intervention, having her thyroid gland removed, thus leading her to hypothyroidism; lower levels of T3 and T4 may have a relationship with the appearance of this ruthless disease.

Over the years, the global medical community has witnessed significant contributions in the field of neuro-oncology. Yet conditions like giant malignant meningioma have remained largely unexplained. Conquering them needs a concerted effort in order to turn the tide against these formidable adversaries.
